# Use and cumulation of evidence from modelling studies to inform policy on food taxes and subsidies: biting off more than we can chew?

**DOI:** 10.1186/s12889-015-1641-5

**Published:** 2015-03-27

**Authors:** Ian Shemilt, Theresa M Marteau, Richard D Smith, David Ogilvie

**Affiliations:** Behaviour and Health Research Unit, Institute of Public Health, Forvie Site, University of Cambridge School of Clinical Medicine, Box 113, Cambridge Biomedical Campus, Cambridge, CB2 0SR UK; Faculty of Public Health and Policy, London School of Hygiene and Tropical Medicine, 15-17 Tavistock Place, London, WC1H 9SH UK; MRC Epidemiology Unit and UKCRC Centre for Diet and Activity Research (CEDAR), University of Cambridge School of Clinical Medicine, Box 285, Cambridge Biomedical Campus, Cambridge, CB2 0QQ UK

**Keywords:** Food, Taxes, Subsidies, Public health, Policy, Mathematical model, Evidence synthesis, Meta-analysis as topic

## Abstract

**Background:**

Food tax-subsidy policies are proposed to hold promise for helping to produce healthier patterns of food purchasing and consumption at population level. Evidence for their effects derives largely from simulation studies that explore the potential effects of untried policies using a mathematical modelling framework. This paper provides a critique first of the nature of the evidence derived from such simulation studies, and second of the challenges of cumulating that evidence to inform public health policy.

**Discussion:**

Effects estimated by simulation studies of food taxes and subsidies can be expected to diverge in potentially important ways from those that would accrue in practice because these models are simplified, typically static, representations of complex adaptive systems. The level of confidence that can be placed in modelled estimates of effects is correspondingly low, and the level of associated uncertainty is high. Moreover, evidence from food tax-subsidy simulation studies cannot meaningfully be cumulated using currently available quantitative evidence synthesis methods, to reduce uncertainty about effects.

**Summary:**

Simulation studies are critical for the initial phases of an incremental research process, for drawing together diverse evidence and exploring potential longer-term effects. While simulation studies of food taxes and subsidies provide a valuable and necessary input to the formulation of public health policy in this area, they are unlikely to be sufficient, and policy makers should not place excessive reliance on evidence from such studies, either singly or cumulatively. To reflect known and unknown limitations of the models, results of such studies should be interpreted cautiously as tentative projections. Modelling studies should increasingly be integrated with more empirical studies of the effects of food tax and subsidy policies in practice.

## Background

Taxes and subsidies imposed on foods, beverages or their component nutrients are proposed to improve health through a simple causal pathway [1-5]. First, they might induce changes in the relative prices of less healthy foods and drinks compared with healthier alternatives. Second, these price changes might incentivise enough people to purchase and consume an overall healthier diet, leading to meaningful reductions in the prevalence of risk factors for non-communicable diseases. Despite considerable uncertainty surrounding both of these propositions, this logic forms the basis of the argument for calls to governments to introduce food taxes and subsidies – especially taxes on sugar-sweetened beverages – as part of broader public health strategies to improve people’s diets [1-5].

Evidence for the effects of food taxes and subsidies on diet-related outcomes derives largely from studies that simulate the potential effects of hypothetical policies using mathematical models (simulation studies). Based on a systematic scoping review [6] and additional targeted searches, there are at least 35 published studies that simulate such effects in High Income Countries [7-41].

There is also direct evidence from a small set of studies that exploit variation between US states in rates of sales taxes imposed on sweetened drinks or snack foods to evaluate their impacts on purchasing, consumption, energy intake or obesity [42-47]. The small effect sizes reported by these studies have been attributed to the low tax rates involved, which vary up to a maximum of around 7% [4,42-47]. These are rare examples of studies that have evaluated food tax-subsidy policies implemented by national, state, or other legislatures in terms of dietary health-related outcomes [4,48]. That such studies are rare is likely to reflect various issues including a lack of policies to evaluate, the short time over which such policies have been sustained, their introduction for reasons other than public health, and a lack of good quality data with which to make suitable comparisons [48].

A third category of study involves experiments conducted in closed laboratory or simulated environments to investigate consumer responses to experimental manipulations of the relative prices of different foods [4,49]. A narrative review of these studies concluded that, whilst price changes can modify purchases of targeted foods, evidence for impacts on the overall nutritional quality of purchases is equivocal [49].

The weight of evidence in this area therefore rests overwhelmingly upon simulation studies. In this article we discuss challenges in the production, synthesis and interpretation of evidence from simulation studies of food taxes and subsidies to inform policy, based on a critical examination of such studies. We argue that the crudeness of food tax-subsidy policies, together with the complexity required in the modelling, renders much of this evidence ambiguous at best, and potentially misleading at worst. Whilst acknowledging the relevance of evidence from other forms of study to the policy debate, we argue that the priority should be to conduct more targeted outcome evaluations of the effects of implemented policies.

## Discussion

### Food tax-subsidy models

Simulation models have an established role as aids to decision making in the initial phases of policy appraisal, to explore untried policy options with uncertain outcomes [50-52]. Such models are intended to represent the essential structure of causal pathways between a policy intervention and changes in outcomes. By definition, all models simplify reality. The degree of simplification is partly a matter of judgement, but is also constrained by the availability of data to inform model conceptualisation^a^ and specification^b^ and to assign values to input parameters – the measurable, quantifiable characteristics incorporated in a model [53-55].

Whilst modelling approaches vary, food-tax subsidy models are typically structured to reflect the simple causal pathway described in the opening paragraph. Food demand systems are estimated in which the tax, subsidy or combined tax-subsidy policy scenario under consideration determines price changes in targeted (taxed or subsidised) foods. The tax or subsidy may be levied directly on one or more specific food categories (for example, a change in the rate of value-added-tax levied on fruits and vegetables [36], or a change in the sales tax levied on sugar-sweetened beverages [14]), or alternatively on the nutrients contained in foods (for example, a subsidy per gram of fibre [36], or a tax per gram of sugar in sugar-sweetened beverages [9]). The foods and nutrients to which taxes and subsidies have been applied in simulation studies invariably appear to be appropriate targets for intervention from a public health perspective. Estimated or assumed price changes in taxed or subsidised foods in turn determine changes in quantities purchased of a set of food products. The sizes of these changes are regulated by own-price elasticities (the estimated change in quantity purchased if the price of that good itself changes), often by cross-price elasticities (the estimated change in quantity purchased if the price of *another* good changes), and by baseline levels of purchasing. These model input parameters are typically estimated by analysis of retrospective large-scale survey data [56-59]. While the set of food products included in these food demand systems is typically wider than those directly targeted by the policy in question, it is still often limited compared with the vast array of foods available in practice. Two examples drawn from each end of this continuum are the food demand system estimated by Kuchler and colleagues, which was limited to own- and cross-price elasticities among four categories of salty snacks [24], and the food demand system estimated by Smed and colleagues, which encompassed own- and cross-price elasticities within and among 23 food groups [36]. Few studies estimate the effects beyond food, however, although in theory this is an important consideration because changes in price could influence overall consumption, and saving, decisions of households. Many models are configured to simulate subsequent changes in quantities of foods consumed (typically assuming a 1:1 or other constant ratio of consumption to purchasing) and corollary changes in energy and nutrient intake. Some extrapolate further still to estimate changes in body weight or body mass index and corollary changes in the prevalence of overweight and obesity [e.g. 12,14,17,37].

Many of the simplifying assumptions incorporated into food tax-subsidy models are reasonable and supported by empirical evidence (for example, the basic assumption that changes in the relative prices of various foods will influence quantities of those foods purchased), or are likely to have negligible influence on estimates of effects (for example, that foods can meaningfully be grouped into categories such as sugar-sweetened beverages, rather than being treated as discrete products such as cola, lemonade and ginger beer). Others could be tested in future studies as published data become available from jurisdictions that have already introduced (and in some cases, subsequently rescinded) relevant policies, such as France, Denmark and Mexico. One example is the ‘pass-through rate’ — a measure of the extent to which a tax or subsidy is passed through to consumers in the form of increased or decreased prices at the checkout. Modellers typically assume this parameter (rate) to be 100%, but uncertainty remains about the influence of potential supply-side responses. These include product reformulation to avoid taxes on specific nutrients or otherwise reduce product cost, the use of countervailing marketing campaigns, or the use of price promotion strategies (e.g. loss leaders or multi-buy deals) to limit (or amplify) the pass-through rate or to mask (or expose) its visibility or salience to consumers [6,58]. Input parameters in food tax-subsidy models are typically described by unique values, and the impact of uncertainty about parameter values on uncertainty in results is therefore not typically addressed. In our view, this is one of the major limitations of such studies that is exemplified in the case of the ‘pass-through rate’ parameter. The routine use of probabilistic sensitivity analyses in these simulation models would not only enable modelling of uncertainty in the pass-through rate to incorporate this uncertainty in the final model outputs (estimates of effects), it would also facilitate examination of the influence of a change in the pass-through rate on these outputs. This would explicitly identify which parameter uncertainty is driving the most uncertainty in model outputs, and these parameters could be prioritised for data collection in future evaluation studies of implemented policies.

A critical weakness in current models is that they are typically static rather than dynamic; they do not incorporate factors such as feedback loops or damping. Feedback loops reflect situations in which initial changes in behaviour may create the conditions for behaviour to change further [60]. For example, public awareness that a product has been taxed because it is unhealthy may further discourage purchasing of that product over and above any effect of the tax-induced increase in its relative price. Damping refers to the capacity of systems to absorb and accommodate change, with the potential to attenuate the effects of policy interventions when these interact with multiple, simultaneously occurring processes [61]. For example, further deregulation of the European Union sugar market in 2017 is expected to further reduce the reference price of sugar in Europe [62]. In the case of taxes that add a percentage to the prices of the taxed product(s), this has the potential to absorb, to some extent, tax-induced increases in the relative prices of foods with added sugars, and therefore moderate any initial effects of a tax on purchasing. The scope for researchers to model these kinds of systems dynamic factors is limited by the lack of relevant evidence to inform corresponding parameter values. However, lack of data should not be sufficient for ignoring conceptually relevant parameters, and deeper uncertainties of this kind that are not quantifiable can still be acknowledged as inadequacies of the models (things we know we have left out or been unable to model properly), alongside unacknowledged inadequacies (things we have not even thought of) [63].

These observations invite a view of simulation studies of food taxes and subsides as preliminary forays in an incremental, phased research process, intermediate in kind between analytic theory and empirical testing [51,53]. From this perspective, effects estimated by simulation studies can be expected to diverge in potentially important ways from those that would accrue in practice.

### Cumulating evidence from food tax-subsidy models

Combining the results of multiple studies, assembled using explicit, systematic methods, can provide more reliable assessments of potential intervention effects than single studies alone [64]. This claim is grounded in notions of science as a cumulative process [65], in which the results of each new study can be integrated with those of existing, comparable studies in an updated, aggregating synthesis, to reduce residual uncertainty about the effects of policy interventions [66,67]. In this section we consider the feasibility of applying three commonly applied aggregative evidence synthesis strategies to cumulate the results of simulation studies of the effects of food tax-subsidy policies: narrative synthesis, statistical meta-analysis and vote-counting. This frames a discussion of whether the results of such studies can meaningfully be cumulated to reduce uncertainty about intervention effects.

#### Narrative synthesis

Published reviews that incorporate evidence from simulation models and other studies (‘see Background’) of the effects of food taxes and subsidies have drawn conclusions broadly in support of their introduction [68-73]. Analyses in these reviews have almost exclusively been limited to narrative synthesis, a textual approach to aggregating evidence from included studies to ‘tell the overall story’ of their findings [74]. Narrative syntheses may be susceptible to conscious or unconscious researcher bias when those telling the story advocate or oppose the policies for which evidence is being synthesised [75-77]. They are also held to be more challenging for larger bodies of evidence [78] and those characterised by a multiplicity of effects that need to be traded off against one another in processing the evidence — both features of the case in point.

#### Meta-analysis

Statistical meta-analysis has been developed and become established in many fields of the health and social sciences. It aims to reduce statistical imprecision and represent uncertainty in estimates of effects by using quantitative techniques to aggregate estimates collected from multiple studies [79]. This involves calculating a weighted average summary effect-size for each outcome along with associated confidence intervals [80]. Procedures for computing study-level effect sizes for continuous outcome variables (e.g. standardised mean differences for measures of food purchasing, consumption or body weight) and inverse variance weights require estimates of mean values of outcomes, associated standard deviations (standard errors for inverse variance weights) and sample sizes [80]. As noted above, most models employed in simulation studies of food taxes and subsidies are deterministic and do not therefore include measures of uncertainty from which standard deviations could be computed. In addition, simulation studies do not have sample sizes, and these cannot typically be inferred due to the same lack of measures of uncertainty. These factors preclude the use of current methods of meta-analysis to synthesise the results of simulation studies of food taxes and subsidies, which explains the lack of published meta-analyses of such studies.

In one systematic review, Eyles and colleagues did, however, derive ‘quantitatively pooled’ estimates of the sizes of modelled effects. This involved calculating descriptive statistics (means and ranges) for own-price elasticities of targeted foods and outcomes, if these had been estimated in three or more included studies targeting the same type of food or nutrient [68]. In practice Eyles and colleagues were able to do this for own-price elasticities (model inputs) of three target products and only two outcome measures. Based primarily on these results, they concluded that “…taxes on carbonated drinks and saturated fat, and subsidies on fruits and vegetables would be associated with beneficial dietary change, with the potential for improved health.” [68]. However, whilst they also reported “substantial variability in outcomes assessed across studies”, the authors did not explicitly reveal the overall large number of outcomes assessed within and across included studies (but just not in three or more studies). In our view, generalizing to ‘beneficial dietary change’ from summary estimates of a handful of outcomes, whilst disregarding hundreds of other outcomes assessed among included studies, may reflect a logical fallacy that is conceptually similar to the selective emphasis that may be placed on some findings over others in a narrative synthesis [76,77].

#### Vote counting

A third candidate quantitative synthesis technique we considered that might be applied to this problem was vote-counting analysis using a hypothesis-testing framework [78]. For each specific outcome, the number of scenarios across simulation studies in which a tax (or subsidy) has been estimated to increase the value of a given outcome would be compared with the number in which a tax (or subsidy) has been estimated to decrease its value. A sign test – a non-parametric statistical test – would then be used to test whether these numbers were different from those expected if the null hypothesis of no effect were true. This basic approach is limited to investigating the presence and direction, but (crucially) not the size, of a potential effect. However, because few specific outcomes have been assessed in multiple simulation studies of food-tax subsidy policies, this would typically have insufficient statistical power to reject the null hypothesis of no difference, leading to the likelihood of false negative results. Alternatively, if we aggregated specific outcomes by the broad construct they capture (e.g. purchasing outcomes for which an increase in value would represent an adverse impact on dietary intake) and applied the same analytic approach, this analysis would be fatally flawed precisely because it investigates the direction but not size of effects. This factor may explain the lack of published syntheses that have utilised this type of analysis.

Consider an illustrative example in which an aggregated set of purchasing outcomes comprises measures of levels of purchasing of (i) sugar, (ii) saturated fats, and (iii) salt. If an individual simulation estimated the potential effects of a food tax on these three specific outcomes as being a large decrease in purchasing of sugar alongside negligible increases in purchasing of both saturated fats and salt, we might reasonably judge that the net balance of potential effects on dietary intake would likely be desirable. Moreover, if twenty studies (using different datasets and variant, reasonable assumptions) were to produce the same pattern of results, then we might reasonably expect this to confer greater confidence in our judgement. However, an aggregate-level vote counting analysis would score this combination of results as 40–20 in favour of undesirable versus desirable effects, with the result of the sign test indicating an undesirable effect on purchasing outcomes. The key implication is that, because vote-counting analyses consider only the direction and not the magnitude of effects, it is not possible to interpret the results of an aggregate-level vote counting analysis as having any bearing on the public health case for or against the introduction of food taxes and subsidies.

Even if a vote counting analysis were preceded by the use of expert judgement to assess whether the overall health impact of a pattern of changes in multiple outcomes is likely to be beneficial or harmful, it may be beyond the cognitive capacity of even the most diligent expert to assimilate and trade off such information in a consistent manner. For example, Table 1 shows modelled estimates of the potential effects of a simultaneous 10% increase in the prices of all foods within three high-fat product categories on purchasing of 32 nutrients, extracted from a single simulation study [8]. The results represent a mixed bag of desirable (e.g. reduction in sugar purchasing or increase in Vitamin E purchasing) and undesirable (e.g. increase in alcohol purchasing or decrease in fibre purchasing) potential effects, with proportionate changes from baseline levels ranging from −5.4% to +2.4% and a degree of variation between ‘modest’ and ‘well-off’ households.

**Table 1:** Predicted effects of a ‘fat tax’-induced 10% price increases in (i) cheese, butter and cream, (ii) prepared meals, and (iii) sugar-fat products* **on quantities of nutrients purchased over a four-week period**

	**Predicted effect**			
	**Modest households**		**Well-off households**	
**Nutrient**	**Direction**	**Size (%)**	**Direction**	**Size (%)**
Energy	↓	−3.6	↓	−3.4
Protein	↓	−3.0	↓	−2.9
Vegetable protein	↓	−5.4	↓	−5.4
Animal protein	↓	−2.3	↓	−2.1
Carbohydrate	↓	−5.1	↓	−5.0
Sugar	↓	−3.7	↓	−3.2
Starch	↓	−6.7	↓	−7.6
Fat	↓	−3.2	↓	−3.1
Saturated fat	↓	−4.5	↓	−4.3
Monounsaturated fat	↓	−3.3	↓	−3.2
Polyunsaturated fat	↑	+0.2	↑	+0.5
Cholesterol	↓	−4.7	↓	−4.5
Alcohol	↑	+2.4	↑	+1.3
Fibres	↓	−3.7	↓	−3.2
Retinol	↓	−2.6	↓	−2.4
Beta-carotene	↑	+0.9	↑	+0.7
Vitamin B1	↓	−4.3	↓	−4.3
Vitamin B2	↓	−3.1	↓	−3.0
Vitamin B3	↓	−2.2	↓	−2.1
Vitamin B5	↓	−2.9	↓	−2.7
Vitamin B6	↓	−3.0	↓	−2.8
Vitamin B9	↓	−2.7	↓	−2.3
Vitamin B12	↓	−0.8	↓	−0.5
Vitamin C	↓	−1.0	↓	−0.8
Vitamin D	↓	−1.6	↓	−1.0
Vitamin E	↑	+1.2	↑	+1.7
Iron	↓	−3.3	↓	−3.2
Calcium	↓	−3.2	↓	−2.9
Magnesium	↓	−3.3	↓	−2.9
Sodium	↓	−5.3	↓	−5.4
Phosphorus	↓	−3.4	↓	−3.2
Potassium	↓	−2.2	↓	−1.9

Source: Adapted from Allais 2010 [8]. *Candy, chocolate, cookies, pastry, ice cream, jam etc.

## Summary

Evidence for the effects of food taxes and subsidies derives largely from simulation studies that investigate the potential effects of untried policies using a mathematical modelling framework. In discussing this evidence base, we have highlighted that effects estimated in this way can be expected to diverge in potentially important ways from those that would accrue in practice. This calls for a basic humility in communicating model results, with clear and consistent acknowledgement that modelled estimates of effects are tentative projections, entirely conditional on incorporated assumptions and data.

We have also set out reasons why results of published simulation studies of food taxes and subsidies cannot meaningfully be cumulated using currently available methods of quantitative synthesis. If these studies are iterative and not cumulative, this implies their contribution is that of providing a series of discrete, exploratory estimates of the potential effects of specific policy scenarios. We acknowledge, however, that other forms of syntheses of studies of food taxes and subsidies might usefully contribute to debate concerning the feasibility, implementation and evaluation of such policies [58,66,81].

Policy makers should therefore not place excessive reliance on evidence from simulation studies of food taxes and subsidies, either singly or cumulatively, in formulating public health policy. Rather, they should be seen as a guide and complement to the development and interpretation of empirical studies of policy options, informing the design of the most robust quasi-experimental studies possible to evaluate actual changes in relative unit retail prices and patterns of food purchasing and consumption, and to allow more accurate estimation of corollary impacts on health-related outcomes [48,82]. Importantly, the data derived from these studies can be used to substantially increase the precision of models to assist in assessing likely generalizability and longer-term effects more robustly than at present, especially in the link between more immediate behavioural endpoints (for example, food, energy or nutrient purchasing) and final health outcomes (principally, mortality and morbidity associated with NCDs) that may be less amenable to direct observation in intervention studies. With the ultimate goal to improve population health, the study of food taxes and subsidies will require modelling to allow integration of evidence from both intervention studies and epidemiological studies, and extrapolation beyond what can be measured from intermediate to final health outcomes [82]. However, these models will only be as good as the data they are based upon. They are therefore not a substitute for well conducted empirical studies, but rather a necessary, albeit not sufficient, component in establishing the evidence for policy in this area. Food tax, subsidy and/or combined tax-subsidy policies will also need to be designed and implemented in close alignment with evaluation planning, reserving the options to reformulate or rescind policies should they fail to achieve desired outcomes (and avoid undesired outcomes) in practice.

## Endnotes

^a^Model conceptualisation is the process of developing an understanding of the real-world causal pathway being modelled and of the potential moderating influences of variant characteristics of the policy itself, the systems in which the policy is implemented, and interactions between the policy and host systems, on outcomes.

^b^Model specification is the process of translating the conceptual model into a mathematical framework.
